# Wild bird captivity in the Brazilian semiarid region: practices, conflicts, and species richness in a protected area buffer zone

**DOI:** 10.1186/s13002-026-00857-w

**Published:** 2026-02-18

**Authors:** Ivã Barbosa, Ricardo Evangelista Fraga, Antonio Iderval Sodré Neto, Cleverson Zapelini, Alexandre Schiavetti

**Affiliations:** 1 Programa de Pós-Graduação em Zoologia – PPGZOO, Universidade Estadual de Santa Cruz – UESC. Campus Soane Nazaré de Andrade, Pavilhão Jorge Amado, Térreo, sala 3007. Rodovia Jorge Amado, km 16 – Bairro Salobrinho. CEP. 45.662-900. Ilhéus, Bahia, Bahia Brazil; 2 Instituto Multidisciplinar em Saúde, Programa de Pós-Graduação em Biociências e Programa de Pós-Graduação em Genética, Conservação e Biodiversidade – PPGECB, Universidade Federal da Bahia – UFBA, Universidade Estadual do Sudoeste da Bahia – UESB. Vitória da Conquista, Bahia, Brazil; 3 Programa de Pós-Graduação em Ecologia e Conservação da Biodiversidade – PPGECB, Universidade Estadual de Santa Cruz – UESC, Ilhéus, Brazil; 4 Laboratório de Etnoconservação e Áreas Protegidas – LECAP, Departamento de Ciências Biológicas, UESC. Ilhéus, Bahia, Brazil; 5 Laboratório de Etnoconservação e Áreas Protegidas – LECAP, Departamento de Ciências Agrárias e Ambientais, UESC. Ilhéus, Bahia, Brazil

**Keywords:** Ethnozoology, Ethno-ornithology, Ethnoecological knowledge, Cultural heritage, Human–bird interactions, Neotropical birds

## Abstract

**Background:**

Keeping wild birds in captivity by rural communities in the Brazilian semiarid region represents a complex blend of cultural heritage and conservation challenges, with birds playing a central role in local traditions and belief systems. Ethno-ornithological approaches offer essential insights into local knowledge systems, including the symbolic meanings attributed to birds and the impact of these practices on wild populations. Such research can inform biocultural conservation strategies that reconcile cultural valuation with biodiversity protection.

**Methods:**

This study examined human–bird interactions in a rural community located in the buffer zone Parnaíba Headwaters National Park in Piauí. Using an emic ethno-ornithological approach, we analyzed the typological categories of bird use, with particular emphasis on captivity by affection. We employed a mixed-method approach to categorize bird use and document participants’ perceptions of human-bird relationships. We compiled a list of species reported by participants, supplemented by rapid sampling.

**Results:**

We identified eight typological categories of bird use, including species commonly captive such as *Sicalis columbiana* and *Brotogeris chiriri*. Historically, local participants reported certain parrot species as food resources. Species from Tinamidae, Icteridae, and Thraupidae were frequently associated with “capture/hunting” practices. Ethnobiological indices indicated high cultural significance for *Ara ararauna*, while several other species showed strong associations with intensity of use. The results reveal how affective, symbolic, and ecological values shape local practices, generating both cultural continuity and conservation tensions.

**Conclusion:**

The Santa Rosa community demonstrates a deep ecocultural connection with local birdlife. The practice of affectionate captivity influences the community’s daily life and underscores the richness of local ethnoecological knowledge. Although primarily driven by emotional motivations, this practice generates critical tension that may jeopardize wild bird population stability and the intergenerational transmission of Traditional Ecological Knowledge (TEK) within the community. These findings contribute to current debates on biocultural conservation, particularly regarding the complex role of TEK in human–avian relationships.

## Background

The interactions between humans and wild birds constitute a complex and multifaceted phenomenon characterized by different conditions, dynamics, and logics of encounters that permeate the history of humanity itself [1]. The plurality of these interactions may have originated in activities such as hunting and the species domestication, which over time shaped distinct perceptions of avifauna [2, 3]. Currently, a variety of sociocultural and ecological factors are considered to influence these processes. Ethno-ornithology, by focusing on the emic perspective, seeks to reveal the complexity of these relationships, moving beyond a utilitarian analysis that permeates the challenges for the conservation of wild birds [4]. Ethno–ornithological studies offer alternatives by addressing these challenges, promoting the decolonization of ornithological knowledge [5–7] and the recognition and application of Traditional Ecological Knowledge (TEK) in biocultural conservation strategies that effectively include local communities in actions and management [8–10].

Due to their ubiquity, wild birds are among the most culturally salient and interactive resources in the daily lives of rural populations in Brazil and Latin America [11, 12]. From these interactions emerge categories of use that, from an ethnobiological perspective, encompass everything from vernacular nomenclature to utilitarian classification patterns tailored to the nature of the resource [13]. Although the categorization of use typologies is an analytical process subject to researcher interpretation, often navigating the tension between overlapping emic dimensions, and standardized scientific frameworks, these classifications remain essential for characterizing the diversity of local resource management [14, 15]. In Brazil, wild bird keeping constitutes one of the most prominent categories of avian use [16–20]. However, the motivations underlying this practice may be associated with a range of contexts that remain poorly explored [21], and studies comprehensively addressing the complex typological categories of bird use by rural communities are even scarcer [22].

Addressing this knowledge gap is particularly urgent in unique sociocultural regions such as the Brazilian Semiarid. This territory covers 12% of the country and is home to 28 million people, 38% of whom reside in rural areas [23]. Its landscape is predominantly defined by the Caatinga biome [24], a distinct biocultural setting characterized by remarkable heterogeneity and a diverse avifauna [25]. Consequently, this region offers a fundamental model for understanding the interplay between biodiversity and human–environment interactions.

Within this context, the avifauna is notably rich, as it is estimated that approximately 591 bird species inhabit the semiarid region, representing about 30% of the 1,971 Brazilian bird species [26]. This is a rich scenario for ethno-ornithological studies, whose percentage reached 4% (in 2011) and 13% (in 2017) in relation to other organisms addressed by Ethnozoology in Brazil [22]. Ethnoornithological research in the Caatinga, despite its growth, is still limited both in comparison with biomes and in its focus on wild bird use, such as hunting, food or commercial resources and illegal traffic, neglecting other dimensions of the human–bird interactions [21, 27]. By engaging local communities, these studies become valuable tools that not only advance scientific knowledge but also ensure the integration of TEK, thus strengthening local participation in natural resource management [27–29].

Against this backdrop, comprehending how rural communities organize, rationalize, and negotiate their interactions with wild birds is crucial for advancing ethno-ornithological theory and informing biocultural conservation strategies within the Brazilian semiarid region. In contexts where protected areas overlap with long-standing cultural landscapes, these interactions are fundamentally shaped by affective bonds, symbolic meanings, and locally embedded ecological knowledge. Such elements may simultaneously support cultural continuity while generating conservation tensions.

In this study, we analyze human–bird interactions within the Santa Rosa rural community, located in the buffer zone of a federal protected area in northeastern Brazil. Adopting an emic perspective, we examine how typological categories of bird use are constructed, hierarchized, and mobilized in everyday life, with particular attention to captivity by affection. Our central research question is: What typological categories of bird use emerge from the community’s daily life? The research objectives of this study are to: examine how typological categories of wild bird use are locally constructed and differentiated; analyze captivity by affection as a culturally embedded form of human–bird relationship; (3) interpreting conflicts and cultural values ​​that arise from local interactions.; and (4) discuss how these interactions reveal tensions between Traditional Ecological Knowledge, cultural heritage, and biodiversity conservation in the Brazilian semiarid region.

## Methods

### Ethics statement

All research participants signed the Free and Informed Consent Term (FICT), agreeing to provide their knowledge while maintaining anonymity regarding personal data and information that could cause harm to themselves or the community if the data were disclosed. Ethical approval for the study was obtained from the Ethics Committee of the Multidisciplinary Institute in Health – Anísio Teixeira, Federal University of Bahia (N° of protocol 71816323.4.0000.5556) and authorization of the Biodiversity Authorization and Information System (Nº SISBIO 89938–1.14/08/2023).

### Study area

**Fig. 1:**
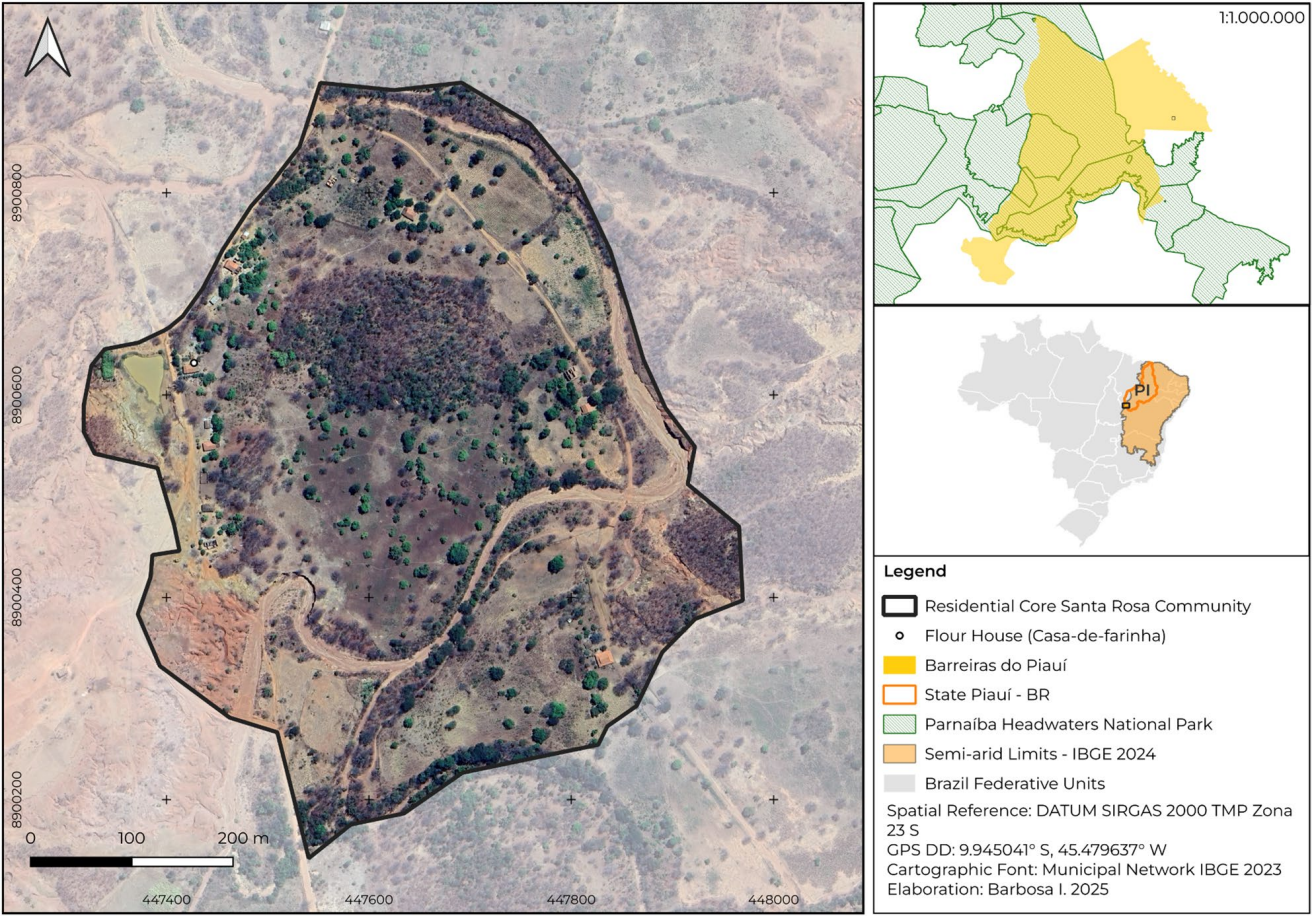
Central nucleus of the Santa Rosa Community – Barreiras do Piauí municipality, Piauí State, Brazil

Our research was conducted in a single rural community named Santa Rosa (9.945041° S, 45.479637° W, Fig. 1), located approximately 3 km from the city center in the municipality of Barreiras do Piauí, Piauí State. The municipality lies within the Brazilian semiarid region, at an altitude approximately of 400 m, with over 50% of its territory bordering Parnaíba Headwaters National Park [30]. The municipality’s population was estimated at 3,264 inhabitants (a population density of 1.51 inhabitants/km²), of which 1,140 live in rural areas [31].

Santa Rosa is a dispersed community, comprising dwellings interspersed with agricultural plots within the Caatinga-Cerrado ecotone. The primary economic activities in the community are raising livestock and subsistence agriculture, prominently featuring the artisanal processing of cassava (*Manihot esculenta*) into flour and its derivatives [32]. The community’s sole flour house (casa-de-farinha) serves as a communal processing hub and a vital space for intergenerational exchange, where ecological knowledge and oral history are transmitted.

### Data collection

Data collection was restricted to the Santa Rosa community. Inclusion criteria required participants to be exclusive residents of the locality and older than 18 years of age. Socioecological and demographic data were recorded based on explicit self-declaration. Ethnicity and religion were recorded only when spontaneously self-identified; otherwise, they were categorized as “undeclared”. Linguistic affiliation was classified according to Glottolog standards.

Prior contact with a local resident facilitated our entry into the community and enabled early interaction with most residents. After this negotiation, a two-day rapport period was conducted, involving immersion and observation of cultural practices. This greatly accelerated rapport-building and minimized the usual challenges associated with access over short periods, following initial consultation with two key informants, a snowball sampling technique was employed [33]. We applied the synchronous and diachronic situation technique to assess the dynamics in participants’ knowledge, through individual and group dialogues, allowing us to track changes in ethnozoological knowledge over time or across different contexts [33, 34]. The initial conversations began with the following guiding questions: (i) Are there many ‘passarinhos’ (local terms for all small birds that fly and sing) here? (ii) Which of these ‘passarinhos’ found here do you know by name? and (iii) Do you do anything to keep the ‘passarinhos’ nearby? From these questions, conversations unfolded as unstructured dialogues focused on local avifauna [35]. Data collection relied heavily on grand-tour questions and non-directive prompts such as “Tell me more about the …?”; “What happens if that bird sings …?”; “Where can I find this species?”; and “How do you recognize male and female of this species?”; among others. This approach offered a transition, allowing informants to freely describe the meaning and uses of birds. without researcher-imposed bias [36, 37]. The data were collected over six consecutive days (July 16–21, 2024), with each interview treated as a sampling unit. Sample size was defined by recurrence and saturation of information [34].

### Data ordering

Data organization involved full transcription of all recorded interviews to ensure detailed understanding of participants’ narratives [35]. The data classification following the criteria: (i) Exhaustiveness of the dataset; (ii) Codification of all contextual and cultural elements relevant to use categories; and (iii) Homogeneity in the identification and standardization of terms or dialogue excerpts that composed the final typological categories of bird use. Excerpts from the participants’ statements complement the descriptive analyses and are presented in single quotation marks and square brackets. Although ethno-ornithological studies often report general categories of bird use [22, 38], those adopted in this study were defined inductively by the participants and are presented in Table 1.

**Table 1:** Use categories of wild birds in the rural community Santa Rosa, Piauí State, Brazil

Use’s Category	Use descriptions	Species number	Use-reports (UR%)*
Captivity by affection (Ca)	Wild birds are raised free-ranging, meaning they are kept outside of cages or enclosures that would confine them.	28	35.9
Non-captive birds (Nc)	Birds that, under some of the local criteria, do not serve for captivity.	38	48.7
Capture/Hunting (CH)	Birds are mentioned as “easy to catch” or “good to hunt”.	13	16.7
Food source (Fs)	Wild birds are mentioned as good to eat.	15	19.2
Benefits (Be)	Birds that are mentioned ecological or pharmacological service.	6	7.7
Prejudice (Pr)	Birds that are mentioned as causing harm to the community.	4	5.1
Commercial (C)	Birds mentioned as a financial purchase-sale object.	12	15.4
Signals (Sg)	Birds that through vocalization/behavior, foreshadow events.	9	11.5

*(UR%) = Use-reports in the category relative to the total number of species cited (*n* = 78, regardless of assigned use)

### Sampling of local birdlife

Ethnospecies were recorded during free-lists to capture the cognitive salience of species (i.e., what are culturally prominent), not necessarily exhaustive biological richness [36]. We also conducted a simple avifaunal survey to compare species recorded through local knowledge with those sampled and to evaluate sampling effort. We selected a protocol suitable for field routine efficiency and cost-effectiveness. Transect sampling included visual records using binoculars (10 × 42 mm) [39], performed by one of the researchers and key-informants, for four hours (starting at 8:00 am), over five consecutive days (July 17–21, 2024), totaling 20 h. Mist-net captures were performed using eight nets (10 m in length x 2.5 m in height; 20 × 20 mm mesh and four bags), arranged in a straight line and rotated daily across three environments with different anthropization levels: a backyard, an understory area, and gallery forest. The mist-nets remained open for nine hours per day on five days (~ 45 h exposure). We calculated the capture effort (E) as the product of the area of a single net (25 m^2^), the exposure time (45 h), and the number of mist-nets (*n* = 8), resulting in a total effort of 9,000 h·m² [40]. After external morphometry and the collection of clinical data (rectal temperature, fecal and blood samples, the fifth primary feather, and ectoparasites), all birds were released at the site of capture. The taxonomy followed the Brazilian Ornithological Records Committee [26]. Furthermore, to better visualize the associations between bird families and the use categories attributed by the participants, a Chord Diagram was generated using the *circlize* package [41].

### Ethnoecological indices and statistical analyses

We adopted both qualitative and quantitative approaches, including phenomenological exploration via analysis narratives, as case study [42]. To assess whether socio-demographic factors influence the local TEK, we employed a Generalized Linear Model (GLM) with a negative binomial distribution (to account for overdispersion in the count data), using the *MASS* and *AER* packages [43, 44]. The response variable was the number of ethnospecies exclusively cited by each participant, and the explanatory variables were age, gender, and education. The education was categorized into two levels: Higher Education/Secondary School (HESS), which includes respondents with higher education, completed secondary school, or secondary school in progress; and Elementary Education/Literacy (EEL), which includes respondents with completed or incomplete elementary school, those who can read and write, and those who can only sign their name.

The following ethnoecological indices were applied: (1) Relative Frequency of Citations (RFC), which reflects the popularity/consensus regarding each species [45], expressed as: RFC = N_s_/N, where N_s_ indicates the number of participants who cited the species regardless of the assigned use, by the total of participants in the research (N); (2) Use Value (UV), where: UV = ΣU/N, where U denotes the total number of citations of the species per typological category of assigned use, and N = total number of participants in the research [46]. Higher UV values indicate a greater intensity of local use, generally associated with specific usage categories. [*e*.*g*., 47, 48]; (3) Relative Importance (RI) is employed to assess cultural significance and versatility, prioritizing species that are widely known by participants and distributed across multiple use categories [*e*.*g.*, *16*, *22*]. The RI was calculated as follows: RI = (NC + CR) × 50, where NC is the number of typological categories attributed to a given species divided by the maximum number of these categories attributed to the most versatile species (those with the highest number of attributed categories); CR is the maximum number of use categories attributed to the most versatile species divided by the total number (*n* = 8, Table 1) of use categories highlighted in the research [46]. Then, we multiplied by the constant 50, for transformation into a percentage scale (%), categorizing the birds into three levels of cultural significance established in the community: low (RI < 41%); medium (41% ≤ RI ≤ 50%); and high (RI ≥ 51%). The indices described were calculated using an Excel Web spreadsheet.

We employed Principal Component Analysis (PCA) to assess patterns and cluster species with similar profiles in relation to the ethnoecological indices. PCA was conducted via the *factoextra* package [49]. Spearman’s test was used to evaluate the correlation between the UV and RI indices.

We estimated sampling sufficiency and species richness using accumulation curves [50], based on the Chao1 (abundance per day) and Chao2 (presence/absence per interviewee) estimators [51, 52]. Calculations were performed with 1000 random permutations, employing the “*estaccum*R()” (Chao1) and “*poolaccum*()” (Chao2) functions from the *vegan* package [53]. The resulting curves, visualized using the *ggplot2* and *patchwork* packages [54, 55], present the mean estimated richness, its 95% confidence interval (CI), and the standard deviation. All primary statistical analysis was conducted using the R software [41].

## Results

### Participants’s socioeconomic profile

The study sample consisted of 16 participants, representing all 17 family units with inhabitants. According to our key informants, the local population is composed of approximately 30 inhabitants (excluding those under 18). This group exhibits a strong kinship structure, with 88.2% of the inhabitants being blood relatives. Regarding ethnocultural affiliation, most participants self-identified as native to the region and “local residents”; however, two self-identified themselves as having Quilombola maternal lineage and one as Indigenous. The sample was predominantly male (75%, *n* = 12; age range: 19–73) compared to female participants (25%, *n* = 4; age range: 18–55). Regarding education, the most frequent level attained was incomplete elementary education (ES); four participants were attending or had completed high school (HS), and only one had pursued higher education (HE). All participants were engaged in family-based subsistence farming, with active involvement of younger household members. At the time of data collection, two participants were retired, three women reported receiving governmental social assistance, and one participant practiced agriculture at a commercial scale (Table 2).

**Table 2:** Socioecological, demographic, and cultural characterization of the Santa Rosa community (Brazilian semiarid region)

Settlement name	GPS coordinates	Altitude (m a.s.l.)	Ecology	Self-defined ethnicity*	Language (Glottolog)	Religion (if declared)	Estimated no. of inhabitants	No. of participants	Gender (*n*)	Age range (years)	Socio-demographic profile
Santa Rosa	9.945041° S, 45.479637° W	~ 400	Cerrado-Caatinga (Brazilian semiarid)	“Moradores locais”/People from here”	Portuguese (port1283)	Undeclared	~ 30	16	12 M 4 F	18–73	Subsistence farmers; family-based agriculture; low formal schooling; occasional social assistance; one commercial-scale farmer

* Ethnicity is reported strictly according to self-identification by community members

Generalized Linear Model (GLM) results showed that none of the tested sociodemographic variables explained variation in bird-related TEK among participants (Table 3). Ethnoknowledge was statistically homogeneous across the sampled population, with comparable response patterns among individuals of different ages, genders, and educational levels. In Santa Rosa, variation in bird-related knowledge was not structured by sociodemographic attributes and was broadly shared among community members, in agreement with ethnobiological surveys conducted in other rural communities of the Brazilian semiarid region [10, 14, 56–59].

**Table 3:** Models adjusted for TEK on birds in the rural community of Santa Rosa, Barreiras do Piauí, Piauí State, Brazil

	Estimate	Std. Error	z value	*P*
(Intercept)	3.51	1.36	2.59	**0.01**
Gender	0.21	0.55	0.38	0.7
Age	−0.03	0.02	−1.22	0.22
Education	−0.48	0.86	−0.56	0.57

### Avifauna and species richness

**Fig. 2:**
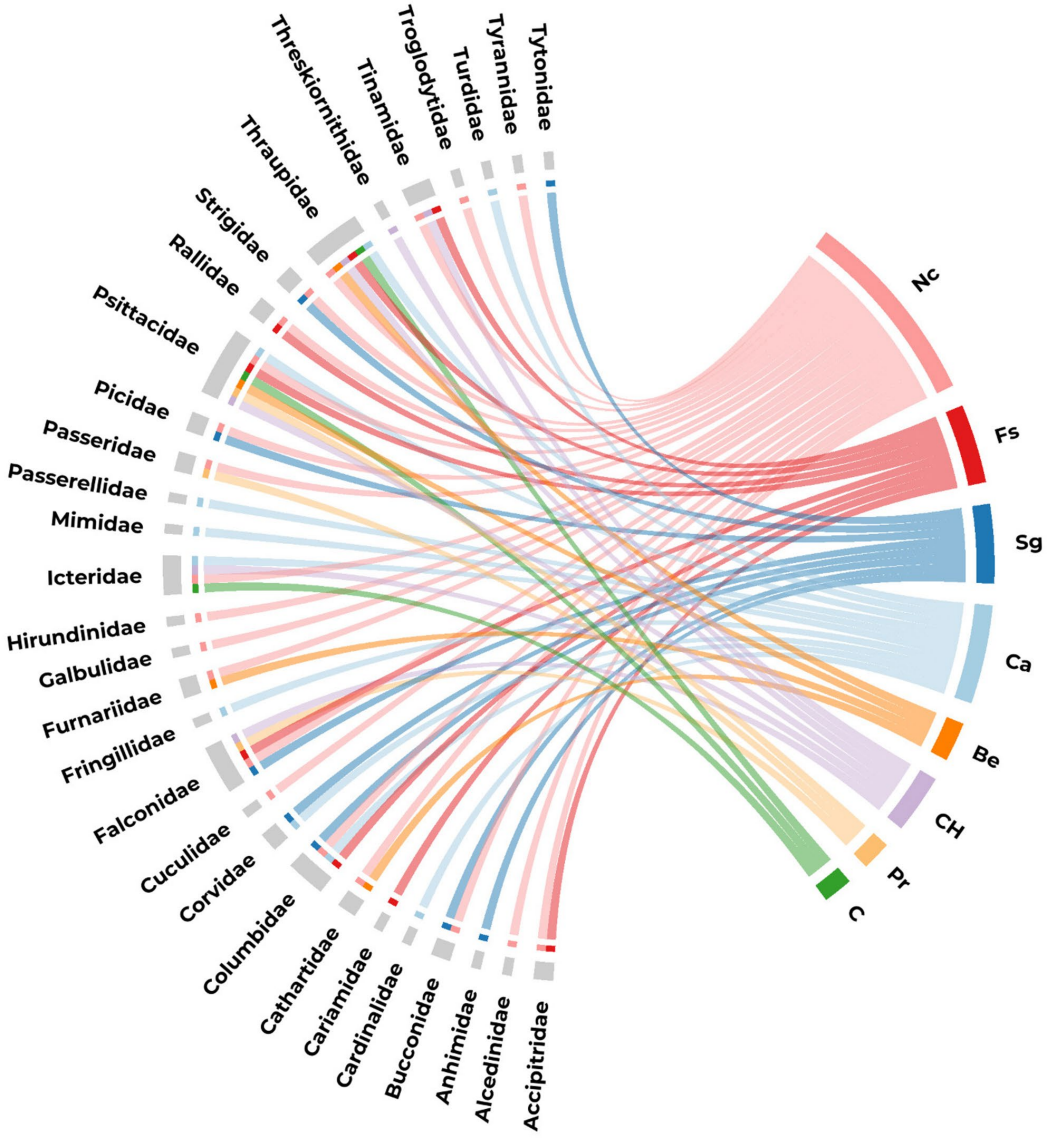
Concentration of use categories for taxonomic families per cited species. Chord diagram used to illustrate associations between bird families (gray, left) and the use categories assigned by participants (colored, right). The thickness of the links indicates the citation frequency of species from a given family in each category. Link colors represent the categories: “Captivity by affection” (Ca), “Non-captive birds” (Nc), “Capture/Hunting” (CH), “Food source” (Fs), “Benefits” (Be), “Prejudice” (Pr), “Commercial” (C) and “Signals” (Sg)

In Santa Rosa, the specific richness comprised 89 bird species belonging to 37 families (Fig. 2), including ethnospecies identified as culturally relevant generic taxa (e.g., “macaws” and “owls”). Among these, 78 ethnospecies were explicitly cited by participants during interviews. Two key informants reported the highest individual richness values (*n* = 32 and 39 ethnospecies). In Santa Rosa, this level of ethnospecies richness falls within the range documented for other rural communities in the Brazilian semiarid region, where ethnobiological surveys have recorded approximately 40–106 bird species associated with multiple use categories [16–22, 56, 60; see also 61].

Thraupidae was the most representative family (*n* = 13 species), followed by Icteridae (*n* = 7), and Psittacidae (*n* = 7), with Columbidae and Picidae each contributing five species. This taxonomic distribution aligns with that documented in ethnobiological surveys conducted in other rural communities of the Brazilian semiarid region, where Thraupidae typically account for approximately 10–15 species per study, Psittacidae for 5–10 species, and Columbidae for 4–8 species [16–20, 57]. All the species were national residents (except *Passer domesticus*). Eight species were endemic to the Caatinga [26]. Four species (*Tinamus tao*, *Penelope jacucaca*, *Celeus obrieni* and *Anodorhynchus hyacinthinus*) are listed as Vulnerable (VU) on the IUCN Red List [62], while *Crypturellus zabele*, *Amazona aestiva*, and *Primolius maracana* are included in Brazil’s national list of threatened species [63]. Detailed information on the taxonomic classification, associated use categories, and ethnobiological index values for all recorded ethnospecies are presented in Table 4.

**Fig. 3:**
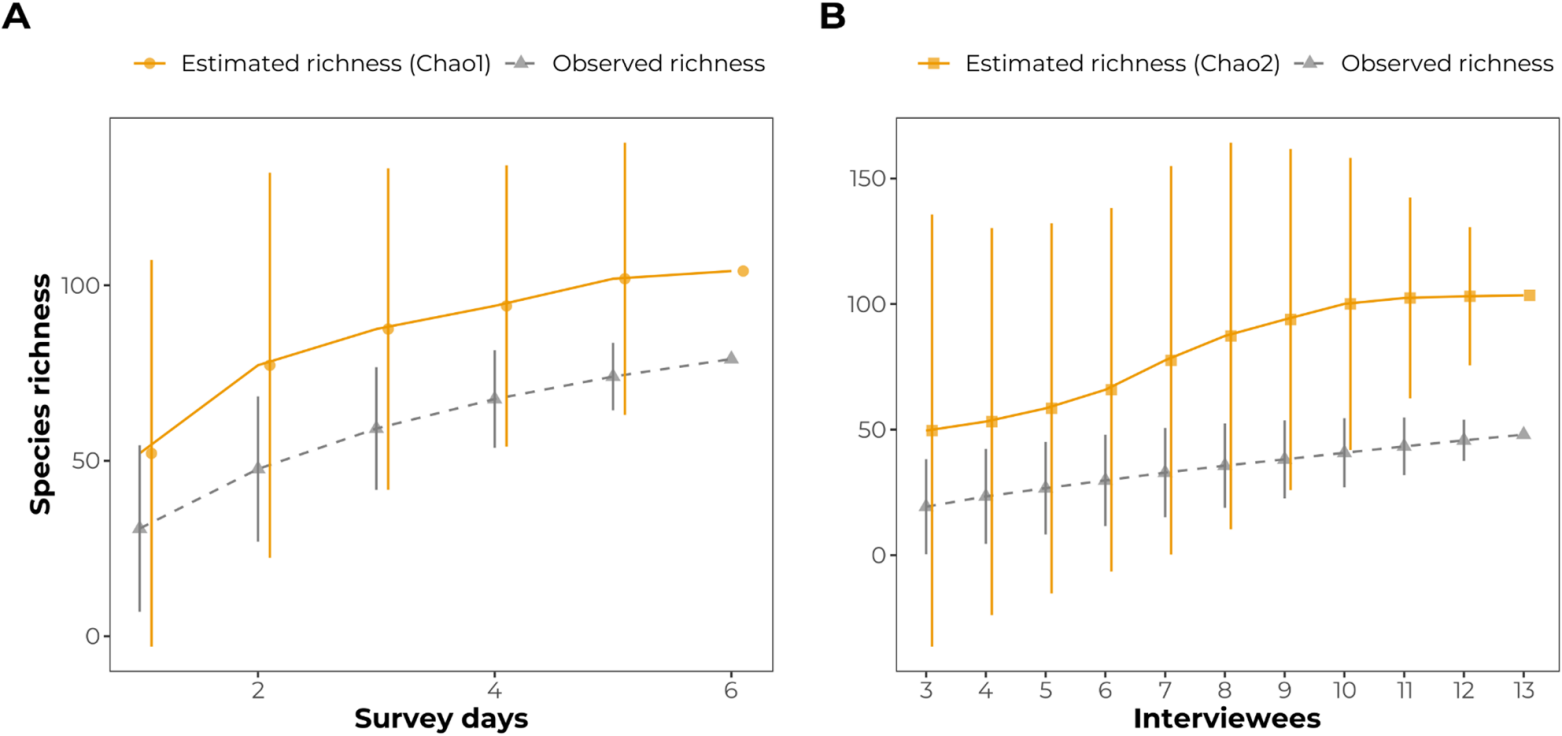
Values richness estimators for bird species – Santa Rosa community, Barreira do Piauí, Piauí State. Chao1 (3 A) and Chao2 (3B) indices. Dashed gray lines and triangles indicate the accumulated observed richness; solid orange lines with circles (3 A) and squares (3B) indicate the estimated richness. Vertical error bars indicate 95% confidence intervals

Species accumulation curves revealed distinct saturation patterns between abundance-based (Chao1) and incidence-based (Chao2) estimators (Fig. 3). Chao1 estimates increased from approximately 52 species on the first sampling day to 104 species by the sixth day, while observed richness rose from approximately 30 to 79 species over the same period. Until the fifth sampling event, confidence intervals remained wide, indicating incomplete saturation of abundance-based estimates. For interview-based data, Chao2 estimates increased from approximately 49 species at the third interview to 103 species by the 13th interview, whereas observed richness ranged from 19.5 to 48 species. The reduction in confidence interval width after the ninth interview and the asymptotic tendencies of the rarefaction curves both indicate a stabilization of estimates for interviews and rapid surveys (Fig. 3). Additionally, free-listing data indicated that participants cited a total of 68 species (Table 4), corresponding to 87% of the recorded ethnospecies, across at least one of the eight use-typology categories.

### The use’ typological categories

A total of 370 citations were recorded in the studied areas, indicating that individual ethnospecies were frequently associated by the participants in multiple use categories, and their respective taxonomic families are represented in Fig. 2. Notably, the categories “Non-captive” (*n* = 38, 48.7% birds use-report – UR), “Captivity affection” (*n* = 28, 35.9% UR), and “Food source” (*n* = 15, 19.2% UR) were the most frequently mentioned in terms of species representation (Tables 1 and 4). Comparable distributions of use categories have been documented in other rural communities of the Brazilian semiarid region. Studies conducted in Caatinga landscapes report that captivity-related uses typically account for approximately 30–45% of cited bird species, followed by food and capture-related categories, a pattern consistent with that observed in Santa Rosa, except for the “Non-captive” category [16–20, 60, 67]. Use Value (UV), reflecting the intensity of local use attributed to individual ethnospecies, ranged from 0.06 to 1.06, while Relative Importance (RI) values varied between 31.3% and 81.3%.

### Ordination of ethnospecies based on ethnoecological indices

**Fig. 4:**
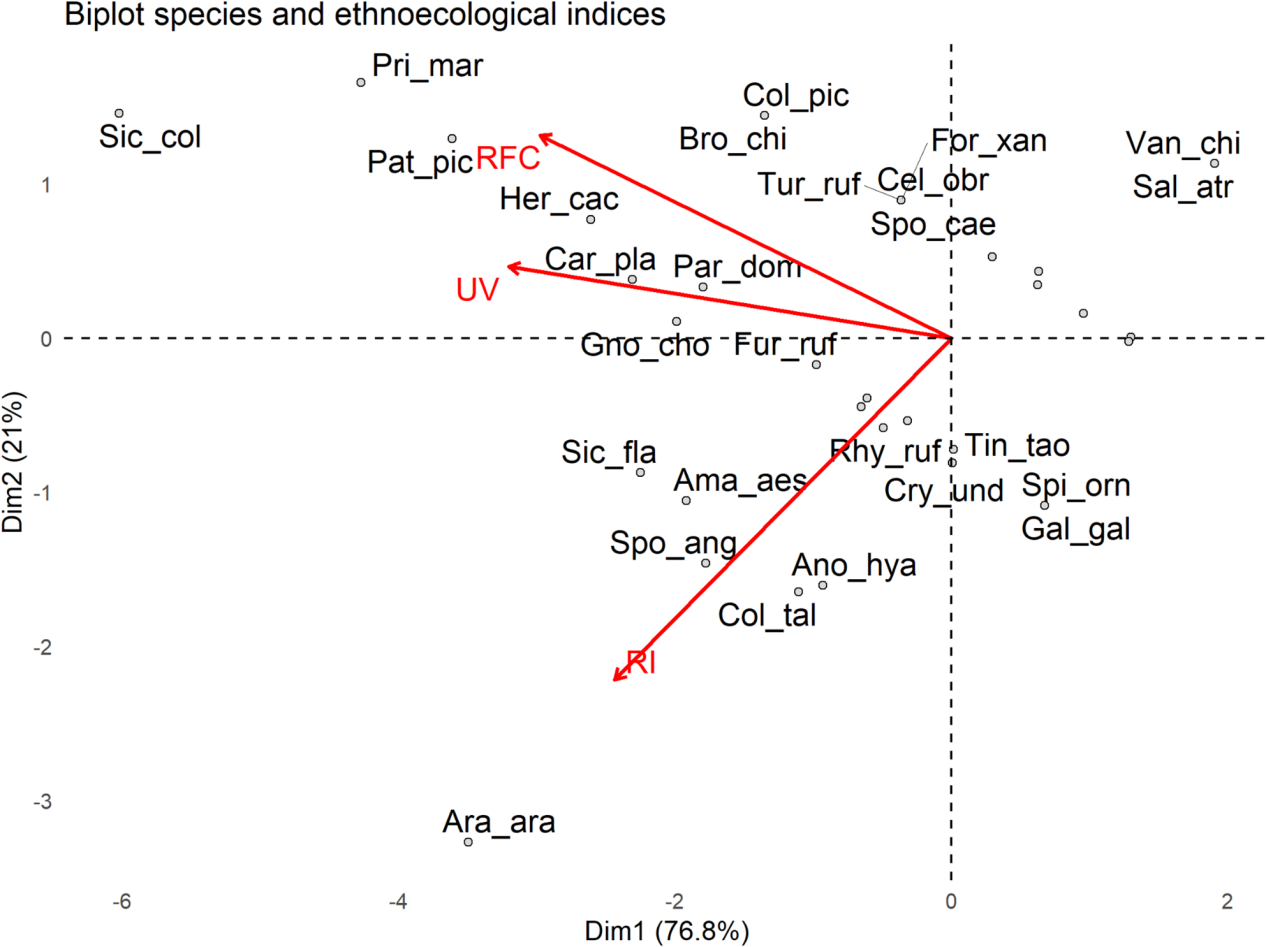
PCA of ethnobiological indices and cited ethnospecies – Santa Rosa community, Barreira do Piauí, Piauí State. Red arrows represent the ethnobiological indices (Relative Frequency of Citations – RFC, Use Value – UV, Relative Importance – RI). Points correspond to ethnospecies (“Gen_spe”) cited by participants

Principal Component Analysis (PCA) summarized the distribution of ethnospecies according to the ethnoecological indices RFC, UV, and RI (Fig. 4). The two main axes (Dim1 and Dim2) explained 76.8% and 21% of the variation, respectively. Similar ordination patterns have been reported for ethno-ornithological studies in northeastern Brazil, where psittacids such as *Amazona aestiva* and *Ara ararauna* consistently present high citation frequencies and high versatility across multiple use categories, despite representing a smaller proportion of total species richness [8, 17, 18, 56, 64]. Species such as *Sicalis columbiana* (22 citations, RFC = 1.4), *Primolius maracana* (16 c., RFC = 1.0), *Patagioenas picazuro* (14 c., RFC = 0.9), and *Herpetotheres cachinnans* (11 c., RFC = 0.7) showed higher RFC values independently of use category. The generic ethnospecies “macaws” combined moderate citation frequency (RFC = 0.6), high versatility of use (RI = 81.3%), and lower use intensity (UV = 0.38). Some species such as *S. columbiana* and *P. maracana*, displayed concordant patterns of high intensity of use (UV ≥ 0.88) and cultural significance (RI ≥ 51.3%). A strong positive correlation was observed between UV and RI (Spearman, rho = 0.66; *p* < 0.05), indicating that ethnospecies with higher cultural relevance also tended to be more intensively used (Fig. 4). The remaining ethnospecies and their raw citation numbers, along with their RFC, UV, and RI values, are presented in Table 4.

### Use’s category captivity by affection

The “Captivity by affection” (Ca) category was cited by 14 participants and accounted for 105 citations distributed across 28 ethnospecies (Table 4). In Santa Rosa, birds assigned to this category were predominantly managed under a free-ranging regime, characterized by the absence of cages or permanent physical confinement. Participants consistently emphasized autonomy of movement and voluntary site fidelity as defining attributes of this practice, expressed through statements such as [‘…we raise them free’], [‘…we don’t keep them in cages’], and [‘…they come and go whenever they want’]. In Santa Rosa, this management pattern reflects a culturally specific interpretation of captivity, in which affective bonds are maintained through proximity and habituation rather than spatial restriction. This contrasts with patterns reported for other rural communities in the Brazilian semiarid region, where affective captivity is more frequently associated with permanent caging, even when companionship is the primary motivation [16–20, 37].

Species associated with this category were predominantly drawn from the families Columbidae, Thraupidae, and Psittacidae (Fig. 2; Table 4). *Columbina talpacoti* (UV = 0.19; RI = 61.3) and *Brotogeris chiriri* (UV = 0.56; RI = 41.3) together accounted for approximately 32% of all citations, indicating their centrality within affective management practices. In regional studies from Caatinga landscapes, these same families recurrently concentrate companionship-oriented uses, reflecting their behavioral tolerance and ecological availability [16–21, 37, 65]. Within Thraupidae, *Sicalis columbiana* (UV = 1.00, RI = 61.3); *S. flaveola* (UV = 0.50, RI = 61.3); and *Paroaria dominicana* (UV = 0.31, RI = 51.3) were the most frequently cited species. Selection criteria for captive species emphasized behavioral traits related to ease of handling and site fidelity, described as [‘.go away’], whereas species with high dispersal tendencies, such as *Sporophila* spp., were less frequently associated with affective captivity despite their valued vocal repertoire. Another relevant finding is that social structure functions as a key exclusion filter across these birds. Species that do not exhibit specific monogamous or solitary (non-gregarious) behaviors were excluded from selection and categorized as “Non-captive”. The affective motivations and management styles in Santa Rosa community have also been documented in other regions of Latin America, where companionship and ease of handling prevail over aesthetic traits such as song complexity [66–68].

The captivity process described by participants followed three distinct and recurrent stages: (i) Extraction of unfeathered nestlings, especially psittacines; (ii) Feeding and rearing of the nestlings; and (iii) The release into backyards under supervision (soft release). The removal of nestlings while still featherless [‘remove them from the nest while still naked (featherless)’] has been widely documented in ethno-ornithological studies across the Brazilian semiarid and other rural regions, where nest extraction is often preferred over adult capture due to lower perceived risk and greater behavioral plasticity of juveniles [16, 17, 19]. Two distinct rearing strategies were described by participants. Manual feeding (traditional): which includes corn or cassava flour, dissolved in hot water and offered with the aid of a syringe into the nestling’s beak (e.g., parakeets/parrots). Comparable feeding practices have been extensively reported for psittacines and small passerines in rural Brazil, where manual feeding constitutes the primary strategy for early survival of nestlings removed from natural nests [17, 19, 20]. A second strategy corresponded to parental or foster feeding under semi-captive conditions. This practice involved wild adults to feed captured nestlings through the construction of cages using “assa-peixe” (*Vernonia polysphaera* B.) lattice, where captured nests of *S. columbiana*, *S. flaveola*, *P. dominicana*, and *Gnorimopsar chopi*, with their nestlings, are placed. Then, a fissure is made between the cage lattices so that the parents have limited access up to the neck to feed them. A study with semiarid rural communities described practices foster-feeding systems alike to those recorded in Santa Rosa, particularly for Thraupidae [*e*.*g.*, *14*, *16*].

Another option that replaces the use of the cage in the rearing strategies for the nestlings *S. columbiana* or *S. flaveola*, was the use of the abandoned nests of *Furnarius rufus*. This nest is collected and subsequently modified with cement or clay to seal the false entrance, and prevent flooding by rainwater; it is then placed on a tree branch near the residence, allowing other birds to naturally feed the nestlings. This practice facilitated interspecific cross-fostering (*e.g*., *S. columbiana* fed by *S. flaveola* with reports of forced interspecific hybridization; and exceptionally of these by *P. dominicana* or *G. chopi* – in situ observation). The ‘feeding’ stage concludes when juveniles achieve foraging independence. Similar post-release variation has been reported for captive-raised birds in northeastern Brazil, where site fidelity is strongly species-dependent [17, 19].

In contexts of “affective captivity,” post-release behavior varies: *Sicalis* spp. often return to the wild but revisit the household [‘…go and after a while return’] and even form mixed flocks, while species like *Patagioenas picazuro* and *Forpus xanthopterygius* typically disperse permanently upon maturity [‘…they leave and don’t return’]. Behavioral plasticity was also recorded, including an interspecific pair bond between a parrot (*Amazona aestiva*) and a parakeet (*Brotogeris chiriri*).

### Use’s category Non-captive

The “Non-captive” (Nc) category does not constitute a utilitarian form per se. However, participants (n = 14) provided significant ethnoecological justifications for excluding these 38 species (84 c.) from “Captivity by affection” (Ca) category. These justifications reflect locally constructed criteria derived from species’ ecological traits, and behaviors. Exclusion by trophic guild: involved species with dietary requirements perceived as difficult to manage or undesirable in captivity. Representative species include: i) Opportunistic omnivores ‒ *Cyanocorax cyanopogon* (UV = 0.25, RI = 51.3); ii) Nectarivores ‒ ‘hummingbirds’ (UV = 0.50, RI = 51.3); iii) Granivores – *Molothrus rufoaxillaris* (UV = 0.38, RI = 51.3); iv) Insectivores ‒ *Nystalus chacuru* (UV = 0.19, RI = 41.3); and v) Carnivores ‒ *Spizaetus ornatus* (UV = 0.13, RI = 51.3). Exclusion by vocalization: some species were excluded due to their unpleasant or undesirable vocalizations. *Pitangus sulphuratus*, *Furnarius rufus*, and *Troglodytes musculus* were explicitly depreciated for their song quality, making them unsuitable for “Ca”. Exclusion by human-wildlife conflict: *Primolius maracana* was excluded based on the “damage” criterion, due to the significant economic loss it causes to crops, particularly corn, leading to a negative perception. Exclusion by husbandry difficulty and dispersal: ethnospecies such as ‘sanhaços’ (*Thraupis* spp.) and ‘sofre/sofreu’ (*Icterus jamacaii*), were described as [‘easy to die’] or [‘hard to raise’]. Participants linked these descriptors to low site fidelity and high dispersal rates, and abandonment of the backyard once individuals reach reproductive maturity, rendering rearing efforts ineffective. Exclusion by social structure: gregarious behavior also functioned as a negative selection filter. Birds referred to as [‘flock birds’], such as ‘currute’ (*Molothrus bonariensis*) and ‘corroxó’ (*Knipolegus franciscanus*) were considered unsuitable for captivity. This observation echoes the selection preference described in “Captivity by affection”, where solitary or monogamous species are favored.

Previous ethno-ornithological studies conducted in rural communities of the Brazilian semiarid region as, Neto et al. [18] registered 21 bird species associated with four main uses (pet, trade, competition, and breeding), with Thraupidae representing more than half of the cited species. Alves et al. [17] reported 197 individual birds belonging to 38 species distributed across 28 families, of which approximately 46% were kept in cages, 21% in vivariums, and only 9% under free-ranging conditions within households or gardens. Similarly, Bezerra et al. [37] observed that 30–45% of cited species were associated with captivity-related uses in Caatinga landscapes. In contrast, in Santa Rosa, the “Non-captive birds” category alone comprised 48.7% of all recorded use-reports. Rather than being inferred from absence in captivity records, non-captive status was explicitly defined by participants based on behavioral and ecological traits, including aggressiveness, high mobility, low site fidelity, and specialized dietary requirements. This quantitative predominance and explicit articulation of exclusion criteria contrast with previous studies, in which a substantial proportion of the local avifauna remains numerically unclassified with respect to non-captivity.

### Use’s category Capture/Hunting

The “Capture/Hunting” (CH) category, comprised 13 ethnospecies (84 c.), corresponding to 16.7% of the recorded avifauna (Table 4). In Santa Rosa, species assigned to this category were locally described as [‘easy to catch’] or [‘good to hunt’], reflecting pragmatic criteria related to accessibility, body size, and encounter frequency. In Santa Rosa, participants established a clear operational distinction between *capture* (live extraction) and *hunting* (lethal extraction), each associated with different motivations and contexts of use. This emic differentiation contrasts with reports from other semiarid communities where bird extraction is described as largely non-selective and not categorically differentiated [60, 64].

Species most frequently associated with this practice belonged to the Tinamidae (*Tinamus tao*, *Crypturellus undulatus*, *Rhynchotus rufescens*, *Nothura boraquira*), and to large psittacines (‘macaws’), which together represented approximately 46% of the species assigned to CH. Quantitatively, the proportion of hunted species in Santa Rosa aligns with values reported with those reported for other rural communities in the Brazilian semi-arid region, where between 14% and 25% of cited avifauna are associated with capture or hunting practices [16, 20]. Alves et al. [17] consider that, although captivity and trade dominate bird-human interactions, hunting remains a recurring practice for a subset of species perceived as abundant and easily accessible.

Despite this correspondence, the relative importance of CH in Santa Rosa was lower than that reported for communities in which subsistence hunting plays a central role. This reduction is reflected both in citation frequency and in temporal qualifiers used by participants, such as references to species that [‘were eaten in the past’]. In Santa Rosa, capture and hunting coexist with, but are secondary to, affective captivity and non-extractive interactions with birds. Such a configuration is consistent with broader regional trends of dietary transition, increased access to alternative protein sources, and regulatory enforcement in protected-area buffer zones [9, 14, 19, 21, 37, 67].

Within the CH category, selective pressure was evident. Species targeted for subsistence were predominantly medium-sized birds with lower tolerance to human proximity, such as *Penelope jacucaca* and *Theristicus caudatus*. Conversely, species like *Caracara plancus* (a predator of domestic chicks and other small birds) and *Primolius maracana* are hunted for conflict mitigation rather than subsistence, a pattern observed in other agricultural interfaces [58, 69–71]. In Santa Rosa, this functional differentiation reinforces a clear separation between extractive and non-extractive relationships with avifauna, as species associated with CH were rarely cited across other use categories (Table 4).

### The hunting

The hunting instruments mentioned by the participants as preferred were the “baladeira” (slingshot) and the ‘shotgun’. Capture aimed at maintaining birds in captivity, often under affective motivations, remains a common practice. In addition to nest collection, participants described several strategies: trap-cages, forming a type of square/rectangular box with a movable lid triggered by a trigger at the bottom of the trap, which contains bait (usually grains, such as canary seeds, *Phalaris canariensis* L.). Comparable trap-based systems have been documented in rural communities of northeastern Brazil, where cage traps account for between 35% and 60% of bird capture events targeting passerines and small columbids [17, 71, 73]. Participants also described the use of live decoys (“lure”) near or inside the trap, an individual of the target species, either with a sophisticated song or a female, used to attract conspecifics, or adding a mirror in the center of the trap to draw individuals by their own reflection, a technique generally applied to more agonistic species such as *P. dominicana*. Trap placement is determined by the target species’ guild: arboreal placement for species like *G*. *chopi*, and terrestrial placement for ground-feeders like *Sicalis* spp.; *Sporophila* spp.; and *Columbina* spp. (frequently captured as bycatch (accidental capture) in ground traps.

From a conservation biology perspective, generalized hunting and pest control practices may disproportionately affect less resilient species, especially in fragmented semiarid landscapes where remnant habitat patches host endemic birds [63]. For instance, hunting and habitat degradation remain documented threats for species such as *Tinamus tao*,* Anodorhynchus hyacinthinus*,* Amazona aestiva*, and *Primolius maracana* (all in categories of uses registered here). These patterns underline the scarcity of studies on participatory co-management for birds in the semiarid region, and the need for integrated biocultural management that reconciles cultural practices with conservation goals [72, 73].

### Use’s category food source

The Food source (Fs) category comprised 15 ethnospecies (34 c.) and corresponding to 19.2% of the recorded avifauna (Table 4). Overall, participants described this practice as predominantly historical, associated with periods of food scarcity rather than current subsistence. Statements such as [‘… good to eat, has a lot of meat …’], [‘was eaten in the past …’], [‘when we were children, our father would take it because sometimes there was nothing to eat at home…’]; [‘even the ‘cadurnê’ was eaten, so we wouldn’t go hungry…’], and [‘… there is no need today, but it was eaten before’] were recurrent, indicating a clear temporal shift from necessity-driven consumption to its near disappearance in contemporary practice.

The most cited species in this category were *Ara ararauna* (UV = 0.31, RI = 81.3), *Anodorhynchus hyacinthinus* (UV = 0.25, RI = 61.3), *Ara chloropterus* (UV = 0.19, RI = 51.3), *Amazona aestiva* (UV = 0.44, RI = 51.3), *Caracara plancus* (UV = 0.25, RI = 51.3), and *Cariama cristata* (UV = 0.06, RI = 41.3). This framing indicates a clear temporal shift from necessity-driven consumption to its near disappearance in contemporary practice, as reported for other rural communities in the Brazilian semiarid region [16, 19, 20]. Although *A. ararauna* exhibited the highest cultural relevance (RI), *A. aestiva* showed the highest intensity of past use (UV), which participants associated with its former regional abundance. Comparable distinctions between cultural salience and consumption intensity have been reported in Brazilian rural communities, where certain psittacids remain culturally prominent even after their decline as food resources [16, 37, 66, 74, 75]. Other ethnospecies are listed in Table 4.

### Use’s category Benefit*s* and prejudice

The combined Benefits and Prejudice (Be–Pr) category comprised ten ethnospecies, representing 12.8% of the recorded avifauna, and accounted for 17 citations across both subcategories (Table 4). This proportion is comparable to values reported for other rural communities in the Brazilian semiarid region, where categories related to benefits and conflict with birds typically represent between 10% and 18% of the locally cited avifauna [16, 19, 37]. The “Benefits” category included six ethnospecies, 7.7% (20 c.) of the recorded avifauna. Species assigned to this subcategory were primarily associated with ecological regulation services, especially scavenging and biological control. A relevant finding is that species such as the ‘vulture’ (*Coragyps atratus*, UV = 0.38, RI = 51.3) and ‘king-vulture’ (*Sarcoramphus papa*, UV = 0.25, RI = 41.3) were most frequently mentioned for their role in deterring crop-raiding birds, especially psittacines such as ‘macaws’ and *Primolius maracana*. Both scavenging species are commonly recognized in Brazilian semiarid landscapes, for their sanitary functions, despite being cited infrequently, fewer than 10% of respondents in comparable studies [19, 56].

Another relevant finding is that participants described a specific management technique associated with this category, consisting of placing a cow’s head (*Bos taurus* L.) on an elevated stake near crop fields, to attract vultures, thereby discourage psittacines. This practice was consistently described as an effective strategy for reducing crop damage without direct interference with the target birds. Comparable non-lethal deterrence strategies have been documented in other semiarid rural contexts, where other species birds perceived as ecosystem regulators are deliberately attracted to mitigate crop damage without direct persecution [16, 37]. Only two participants explicitly associated “Benefits” with ecosystem services, particularly seed dispersal [‘birds spread seeds, they eat a fruit here, pass it in the feces, and leave it ahead’]. Nevertheless, species such as *Sporophila angolensis*, *Furnarius rufus*, and “macaws” were included in this category (Table 4). A single medicinal use was recorded within the community, attributed to woodpeckers (Picidae), whose skull is toasted and prepared as an infusion for the treatment of headaches. Therapeutic uses of woodpeckers are consistently rare in ethnobiological surveys conducted in northeastern Brazil, usually accounting for less than 5% of recorded bird uses [14, 76].

The “Prejudice” (Pr) category accounts for four ethnospecies (25 c.). *P. maracana* (UV = 0.88, RI = 51.3) was the most frequently mentioned, accounting for 56% of reports (*n* = 14), primarily due to the damage it causes to corn (*Zea mays* L.) and sesame (*Sesamum indicum* L.) crops. Crop-raiding psittacines are similarly identified as major sources of economic loss in other Caatinga communities, where conflict-related citations frequently exceed 30% of use reports [16, 37, 73]. *Ara ararauna* was also included in this category, although participants did not specify the extent of crop damage, while *Caracara plancus* was cited for predation on domestic chicks and small birds in household yards. In all cases, species assigned to the “Prejudice” category were framed as sources of economic or domestic loss, a pattern commonly reported in small-scale farming systems [56, 69].

### Use’s category commercial

The “Commercial” (C) category was cited by six participants, encompassing 12 ethnospecies (24 c.). This category represented approximately 15% of the ethnospecies recorded in Santa Rosa, a proportion consistent with values reported for other rural communities in the Brazilian semiarid region, where commercial use of birds generally ranges between 10% and 25% of locally cited avifauna [17, 18, 21, 37]. According to participants, bird trade occurs primarily in the nearby city of Gilbués, where certain species are sought as adult breeding stock for captivity. The species of the families Thraupidae and Icteridae were the main targets, particularly *S. columbiana*, *S. flaveola*, *Sporophila angolensis*, *S. nigricollis*, *P. dominicana*, and *I. jamacaii*. These taxa in Thraupidae and Icteridae are widely reported in regional studies as preferential targets of trade due to attributes such as song performance, coloration, and ease of maintenance in captivity, from frequently account for more than 60% of traded bird species [17, 18, 21]. A recurrent account across participants involved the case of ‘xuré’ (*S. columbiana*), stating that the species was originally absent from the Santa Rosa community, regarding the species’ introduction. According to local narratives, a breeding pair was acquired through trade, raised in captivity, and subsequently released, leading to the establishment of the species in the locality.

Participants also reported that commercial activities previously occurred within the community itself, particularly between 20 and 30 years ago. During that period, species such as *Sarcoramphus papa* and *Anodorhynchus hyacinthinus* were reportedly sought after for purchase. Participants reported that these activities have largely declined following the establishment of the federal protected area and the increase in inspection and enforcement actions. Comparable temporal declines in local bird trade following the establishment of protected areas and increased surveillance have been documented elsewhere in the semiarid region, where trade persists mainly in urban centers rather than within rural communities [21, 37, 67].

### Use’s category signals

The Signals (Sg) category was indicated by eight participants (40 c.), and comprised nine ethnospecies (Table 4). Participants consistently described these signals (ornitho-augurs) as part of long-standing local knowledge transmitted across generations, as following: i) Funereal signals – announcement of death for ‘verdadeira/asa-branca’ (*Patagioenas picazuro*, UV = 0.50, RI = 51.3), ‘difunteira/rolinha-branca’ (*Columbina picui*, UV = 0.56, RI = 41.3), ‘rasga-mortalha’ (*Tyto furcata*, UV = 0.25, RI = 41.3) and ‘mãe-da-lua’ (*Nyctibius griseus*, UV = 0.13, RI = 41.3). This category represented approximately 11.5% of the recorded ethnospecies, a proportion lower comparable to those reported in other ethno-ornithological studies conducted in areas of the same semiarid region, where symbolic or omen-related uses typically involve plus 15% of cited bird species [11, 14, 77]. This typology reflects the symbolic significance of birds, whose vocalizations or behaviors are interpreted as indicators of social events, weather patterns, or omens.

Similar associations between owls, doves and death-related omens have been reported across rural communities of northeastern Brazil, where between 20% and 35% of respondents mention such species as funereal or ominous signals [11, 14]. The associations were described through specific vocalizations or nocturnal behaviors perceived as threatening. ii) Societal and environmental signals – announcement of visits from friends or close relatives if the bird flies into the house, hovers in the living room and trills for hummingbirds (*Campylopterus* sp.) and ‘acauã/cauã’ (*Herpetotheres cachinnans*). Participants emphasized these occurrences as routine indicators within community life, and they classify three different songs by ‘acauã/cauã’: (1) As a harbinger of rain; (2) For unwanted visitors; and (3) Announcing the death of close relatives. These distinctions were widely agreed upon across interviews and consistently referenced as reliable cues for interpreting environmental or social changes. Comparable classifications of multiple vocal patterns attributed to a single species have been documented in other semiarid communities, where raptors and nocturnal birds concentrate a high diversity of symbolic meanings and account for up to 40% of all omen-related citations [14, 77].

### Ethnoecological perceptions

Participants demonstrated a detailed body of ethnoknowledge concerning local avifauna, encompassing biological, ecological, and behavioral attributes. Ethnoecological perceptions were reported by all participants and involved patterns of abundance, population change, seasonality, diet, reproduction, and morphological differentiation, directly informing use categories and conflict attribution. Regarding perceived population trends, 13 participants (~ 81%) stated that birds remain abundant in the community, although declines were explicitly reported for 10 species (12.8% of the recorded avifauna). Species perceived as declining included *Crypturellus undulatus*, *Penelope* jacucaca, *P*. *superciliaris*, *Leptotila verreauxi*, *Sarcoramphus papa*, *Vanellus chilensis*, *Celeus obrieni*, *Sicalis flaveola*, and *Paroaria dominicana*. Tinamous (*Nothura* spp.) and partridges (generic local term) were consistently described as “disappeared.” These perceptions were emically associated with past external harvesting, habitat loss, fire, and historical hunting pressure, as expressed in statements such as: [‘people from outside used to come here to catch birds.’]; [‘ … even the ‘cadurnê’ is no longer seen here, only up there, towards the swamp’]. Alves et al. [17] and Bezerra et al. [37] point to comparable patterns of perceived population decline linked to habitat disturbance and external extraction have been reported for Caatinga communities, where 10–25% of locally known species are described as decreasing or locally absent [56]. Bezerra et al. [67] explained that species sensitive to habitat alteration and hunting pressure are often the first to be perceived as declining by local populations, reinforcing the reliability of emic population assessments. Conversely, four species (5.1%) were perceived as increasing locally: *Sporophila angolensis*, *Icterus jamacaii*, *Nystalus maculatus*, and *N. chacuru*. Participants explicitly linked these increases to free-ranging affective captivity practices and to the proximity of the federal protected area, which was described as enhancing local bird richness. Similar associations between protected areas and perceived increases in generalist or synanthropic species have been documented in northeastern Brazil [14, 28, 29, 48].

Seasonality emerged as a central ethnoecological axis. Participants identified the dry season as the most critical period for birds, while the rainy season was associated with higher abundance due to fruit availability. During this period, species most frequently cited as abundant included *Thraupis sayaca*, *Icterus jamacaii*, *I. pyrrhopterus*, *Ramphocelus bresilia*, *Sicalis flaveola*, *Primolius maracana*, and *Sporophila* spp. Increased reproductive activity during the rainy season was associated with *Columbina* spp., *Furnarius rufus*, and *Nystalus maculatus*, attributed to the availability of wet clay for nest construction.

Dietary knowledge, birds feeding on insects, fruits, or mixed diets, reinforced criteria for selecting species for captivity. In time of the abundance of fruit’s “buriti-palm” (*Mauritia flexuosa* L.f.), “mango” (*Mangifera indica* L.), and “cashew” (*Anacardium occidentale* L.), attracts more birds, with psittacids as the most frequent species on “buriti-palm”. Comparable dietary classifications guiding bird keeping and valuation for semiarid communities were observed by Neto et al. [18], and Bezerra et al. [37] where fruit availability is a key ecological cue shaping human–bird interactions.

Sexual differentiation was primarily identified through body size and vocalization [‘The females do not sing, and only those of some species accompany the male’s song’]. Participants reported females as generally smaller than males, and males as the primary singers. Notably, four elderly male participants (65–73 years) identified sexual dimorphism in *Gnorimopsar chopi* based on tongue coloration, with males presenting darker pigmentation. All participants distinguished juveniles from adults by plumage characteristics.

## Discussion

Taken together, the results reveal that bird–human interactions in Santa Rosa are not structured around isolated uses or discrete categories, but rather emerge from an integrated cultural system in which affective, symbolic, utilitarian, and regulatory dimensions coexist and overlap. Rather than reflecting opportunistic or random practices, the recorded use categories express shared cultural logics, emic selection filters, and historically accumulated ecological knowledge that shape how birds are perceived, managed, tolerated, or excluded within the landscape. The relevance of these findings therefore lies less in species richness per se than in the cultural processes that shape knowledge transmission, captivity decisions, conflict management, and perceptions of environmental change. From this perspective, the results offer insights into how biocultural dynamics influence conservation outcomes in semiarid landscapes.

### Traditional ecological knowledge and Sociocultural aspects

The relative uniformity of bird-related knowledge observed among participants indicates a high degree of shared Traditional Ecological Knowledge (TEK) within the Santa Rosa community. Although demographic attributes such as age or formal education are often expected to structure variability in ethnobiological knowledge [60], the empirical patterns observed here indicate that daily engagement with birds and routine environmental interactions outweigh such distinctions.

A complementary, non-exclusive explanation relates to sample size, which may limit the statistical detection of subtle intra-community differences [e.g., *58*]. Nevertheless, the stabilization observed in rarefaction curves should not be interpreted as evidence of exhaustive sampling of local avifauna, but rather as an indication of redundancy in culturally shared knowledge. In interview-based ethnobiological contexts, this pattern emerges when information is widely distributed and repeatedly cited across participants, particularly in communities characterized by strong oral transmission and collective daily practices [36].

Even under these constraints, the internal coherence and quantitative consistency of the reported TEK support its interpretation as a culturally stabilized system rather than a fragmented or idiosyncratic body of knowledge. This homogeneity reflects continuous sociocultural transmission occurring in shared spaces of interaction, such as households, backyards, and communal production areas. Comparable dynamics have been described in rural communities of the Brazilian semiarid region, where orality and daily practice reduce individual variability in knowledge transmission [58–60], and where environmental predictability and sociocultural continuity reinforce one another [e.g., *14*, *18*, *56*].

From a conservation perspective, these same sociocultural processes, central to community life and the maintenance of TEK, also underpin socio-biocultural sustainability. In buffer zones of protected areas, such as the context surrounding Santa Rosa, the persistence of shared knowledge systems may play a critical role in mediating human–environment interactions and supporting locally grounded conservation strategies [60, 63].

### Captivity by affection as a cultural system

The use related-Captivity in Santa Rosa cannot be interpreted solely as an extractive or commercial practice. Instead, the results indicate that captivity for affection constitutes a culturally embedded system grounded in emotional bonds, aesthetic appreciation, and long-term coexistence with birds within domestic spaces. This form of captivity is regulated by emic selection filters that prioritize song, temperament, and adaptability to household environments, although it may also evoke ethical concerns regarding animal welfare and commodification [48, 56, 58, 60]. These filters explain why certain taxa are repeatedly favored, while others remain non-captive or are actively avoided. The captivity in Santa Rosa does not operate in isolation, but intersects with other use categories, including symbolic meanings, occasional trade, and conflict mitigation. From an analytical perspective, this system challenges simplistic conservation narratives that frame captivity exclusively as illegal extraction [66, 71, 78–81]. Instead, it highlights the need to distinguish between large-scale commercial exploitation and practices that are culturally rooted in affective relationships.

The category of non-captive birds reveals a critical emic distinction between birds that are culturally tolerated and those that are actively incorporated into human spaces. Non-captivity does not imply ambiguous use category; rather, it reflects moral, symbolic, or practical boundaries that regulate human–bird interactions. This finding in the Santa Rosa community, underscores the importance of emic categorizations, which do not map neatly onto etic conservation classifications but nonetheless shape interaction intensity and extraction pressure.

#### Symbolic dimensions and methodological limits

Symbolic uses, particularly those related to signals and omens, reveal that birds occupy a role that extends beyond material utility. These associations structure interpretations of social events, environmental change, and uncertainty. Overall, the distribution of the symbolic bird uses recorded in Santa Rosa aligns with regional patterns, which are concentrated in a restricted set of taxa, predominantly Columbidae, Strigiformes, and Caprimulgiformes, exhibiting locally specific classifications and interpretive nuances [11, 14].

The present study does not attempt to instrumentalize symbolic knowledge but rather to document its persistence and internal coherence. Recognizing these limits is essential to avoid reducing symbolic relationships to functional proxies. While such dimensions are culturally robust, they remain methodologically challenging to translate into conservation frameworks that prioritize measurable outcomes.

#### Conflicts and cultural values

Human–bird conflicts in the Santa Rosa community are not uniformly distributed across the recorded avifauna, but are concentrated in a restricted set of ethnospecies and use categories. Of the 78 ethnospecies documented, 10 species (12.8%) were explicitly associated with conflict-related perceptions, primarily within the Prejudice (Pr) category and secondarily across Commercial (C), Capture/Hunting (CH), and Benefits (Be). Regional and national datasets indicate that captivity related-uses of passerines (particularly from the families Thraupidae, Emberizidae, and Icteridae) and psittacines constitute major pressures on avifauna in the Brazilian semiarid region [17, 37, 78]. Some of the culturally ingrained preference criteria, such as those reported for *Sicalis* spp., *Sporophila* spp., *Paroaria dominicana*, and *Icterus jamacaii*, make these birds even more attractive for the live pet trade [18, 27]. For instance, the saffron finch (*S*. *flaveola*) was found in all 23 studies in regions of Brazil, totaling 39,781 birds seized over a 12-year period [82]. However, these large-scale data contrast with the pattern observed in Santa Rosa, because the Commercial-related use category takes the form of occasional purchase/exchange, focused on possession and cultural value, rather than large-scale external trade.

Ethno-ornithological studies conducted in rural communities of the Caatinga commonly indicate Psittacidae species kept in captivity [8]. The majority (37%) of the birds were kept in cages or aviaries, and only 9% (total 197 birds reported) were free within the homes or gardens of the breeders [17, 37]. This trend highlights the scale of local demand and aligns proportionally with the national pattern, representing 12% (approximately 25) of the 411 Psittacidae species, including those of the genera *Amazona*, *Ara*, and *Brotogeris* recorded here, traded as pets (Table 4) [27]. Alves et al. [8] and Destro et al. [64], using ethnobiological indices, also locally documented high multifunctionality and high citation frequencies for Psittacidae of this species throughout northeastern Brazil.

While hunting-related uses account for about 30% of the species mentioned [19], the lower representation in Santa Rosa suggests a reconfiguration of the conflict: from extraction driven by subsistence to tensions more related to crops and domestic problems. For instance, the use and capture of wild birds in Santa Rosa communities, such as *Columbina* spp. similarly suggesting a possible standard of use for such species [56].

The multifunctionality of some species, such as *P*. *maracana* and *C*. *plancus* (Table 4), amplifies the perception of conflicts (negative perceptions coexist with strong cultural salience), and generates local pressures on them. Similar overlaps between high cultural value and conflict have been reported for rural communities in the Brazilian semiarid region, where problem species often account for 30–40% of negative citations, even when simultaneously assigned to captivity, symbolic, or commercial categories [14, 16, 37, 58]. However, in Santa Rosa the positive effect of indirect non-lethal strategies in managing agricultural conflicts proved to be culturally positive, contrasting with studies in other areas of the Caatinga, where scavenging birds are rarely actively mobilized to protect crops [19, 37].

#### Conservation, conflict, and cultural ambiguity

Human–bird conflicts in Santa Rosa are not uniformly negative but are characterized by cultural ambiguity. Certain birds are simultaneously valued, and perceived as problematic, depending on context. Crop damage, poultry predation, and perceived nuisance coexist with symbolic significance or utilitarian recognition. The description of a unique ecological tool, the use of vultures as deterrents to birds attacking cultivated areas, represents a local practice (emerging from cumulative traditional knowledge), that constitute a novel, non-lethal ethno-ecological management strategy for addressing persistent conflicts caused by Psittacines; a hypothesis to be tested.

The predominance of utilitarian and affective criteria in bird use decisions creates localized demand that is culturally structured rather than market-oriented. In this context, the emphasis on tractable, abundant, and behaviorally tolerant species may indirectly reduce pressure on rarer taxa, while simultaneously increasing the exploitation of a limited subset of species repeatedly selected for captivity or trade. Such dynamics are consistent with a target-shifting process, in which cultural preferences and availability guide substitution rather than species rarity per se [66, 73, 81]. In Santa Rosa, this pattern is reflected in the recurrent use of species that combine cultural value with ecological tolerance, suggesting that pressure is redistributed within the avifauna rather than eliminated. This process illustrates how culturally mediated selection filters can simultaneously buffer and intensify conservation pressures, depending on species traits and social demand.

The results show that conflict is not evenly distributed across taxa but concentrates on culturally salient species that intersect multiple use categories. This multifunctionality increases both tolerance thresholds and the risk of persecution, depending on situational factors. Such ambivalence highlights the limitations of conservation strategies that fail to engage with culturally grounded perceptions of harm and benefit. The conservation initiatives in contexts such as Santa Rosa must engage with culturally embedded systems of knowledge and use rather than treating human practices as external threats [*see* *16*, *19*]. The coexistence of affection-driven captivity, symbolic valuation, and conflict management suggests that conservation success depends on negotiating cultural meanings rather than enforcing categorical prohibitions.

Integrating local TEK into conservation planning does not imply uncritical validation of all practices, but it does require acknowledging that culturally normalized behaviors shape real ecological outcomes. In this sense, Santa Rosa illustrates how biocultural conservation must operate at the intersection of cultural continuity, environmental change, and negotiated coexistence.

**Table 4:** Avifauna in the Santa Rosa community, surrounding Parnaíba Headwaters National Park – Barreiras do Piauí – Piauí State

Taxonomic Classification	Local Name	Sampling	TNC	Use’s Categories	Indices		
Order - Family - Species					RFC	UV	RI
Tinamiformes							
**Tinamidae**							
*Tinamus tao* ^VU^	Nambu	I	4	Fs, CH	0.1	0.06	41.3
*Crypturellus undulatus*	Jaó ^d^	I	2	Fs, CH	0.1	0.25	51.3
*Crypturellus zabele * ^EN^	Zambelê	I	1	0	0.1	0	31.3
*Rhynchotus rufescens*	Perdiz ^d^	I	4	Fs, CH, C	0.1	0.25	51.3
*Nothura boraquira*	Cadurnê ^d^	I	5	Fs, CH	0.3	0.31	51.3
Anseriformes							
**Anhimidae**							
*Anhima cornuta*	Anhuma	I	1	Sg	0.1	0.06	41
Galliformes							
**Cracidae**							
*Penelope superciliaris*	Jacu ^d^	I	6	Fs, CH	0.2	0.13	41.3
*Penelope jacucaca * ^VU^	Jacupemba ^d^	I	2	Fs, CH	0.2	0.38	51.3
Columbiformes							
**Columbidae**							
*Patagioenas picazuro*	Verdadeira	I, AV, MN	14	Ca, Sg	0.9	0.5	51.3
*Leptotila verreauxi*	Juriti ^d^	I, AV	1	Fs	0.1	0.06	41.3
*Columbina talpacoti*	Rolinha-roxa	I, AV, MN	3	Ca, Fs, Nc	0.2	0.19	61.3
*Columbina squammata*	Fogo-pagou	I, AV, MN	2	Nc	0.1	0.13	41.3
*Columbina picui*	Difunteira	I, AV, MN	9	Sg	0.6	0.56	41.3
Cuculiformes							
**Cuculidae**							
*Crotophaga ani*	Anu-preto	I, AV, MN	1	Nc	0.1	0.06	41.3
*Guira guira*	Anu-branco	I, AV	1	Nc	0.1	0.06	41.3
Nyctibiiformes							
**Nyctibiidae**							
*Nyctibius griseus*	Mãe-da-lua	I	2	Sg	0.1	0.13	41.3
Apodiformes							
**Trochilidae**							
*Eupetomena macroura*	Tesourão	AV, MN	–	–	–	–	–
*Phaethornis pretrei*	Limpa-casa	I, AV	4	Nc, Sg	0.3	0.25	51.3
*Campylopterus sp.*	Beija-flor	AV, MN	–	–	–	–	–
Gruiformes							
**Rallidae**							
*Gallinula galeata*	Galinha-d’água ^d^	I	1	Fs, CH	0.1	0.06	41.3
Charadriiformes							
**Charadriidae**							
*Vanellus chilensis*	Quero-quero ^d^	I, AV	2	0	0.1	0	31.3
Pelecaniformes							
**Threskiornithidae**							
*Theristicus caudatus*	Curiaca	I	2	CH	0.1	0.13	41.3
Cathartiformes							
**Cathartidae**							
*Sarcoramphus papa*	Urubu-rei ^d^	I	4	Se	0.3	0.25	41.3
*Coragyps atratus*	Urubu	I, AV	4	Nc, Se	0.3	0.38	51.3
*Cathartes burrovianus*	Urubu-cabeça-amarela	AV	–	–	–	–	–
Accipitriformes							
**Accipitridae**							
*Spizaetus ornatus*	Gavião-penacho	I	1	Fs, Nc	0.1	0.06	51.3
Strigiformes							
**Tytonidae**							
*Tyto furcata*	Rasga-mortalha	I, AV	4	Sg	0.3	0.25	41.3
**Strigidae**							
Generic citation	Corujas	I	3	Sg	0.2	0.13	41.3
*Glaucidium brasilianum*	Caburé	I	1	Nc	0.1	0.06	41.3
Coraciiformes							
**Alcedinidae**							
*Megaceryle torquata*	Martim-pescador	I	1	Nc	0.1	0.06	41.3
Galbuliformes							
**Galbulidae**							
*Galbula ruficauda*	Beija-flor-grande	I, AV	1	Nc	0.1	0.06	41.3
**Bucconidae**							
*Nystalus maculatus*	Pira-rosa ^i^	I, MN	4	Nc	0.3	0.13	51.3
*Nystalus chacuru*	Galo-de-vovô ^i^	I	3	Nc	0.2	0.19	41.3
Piciformes							
**Picidae**							
*Melanerpes candidus*	Pinica-pau-branco	I	2	Nc	0.1	0.6	41.3
*Campephilus melanoleucos*	P.-cabeça-vermelha	I, AV	2	Nc	0.1	0.13	41.3
*Celeus flavus*	Pinica-pau-amarelo	I	2	Nc	0.1	0.06	41.3
*Celeus obrieni * ^VU^	Pinica-pau ^d^	I	6	Nc	0.4	0.38	41.3
*Colaptes campestris*	Tange-veado	AV, MN	1	Nc	0.1	0.06	41.3
Cariamiformes							
**Cariamidae**							
*Cariama cristata*	Seriema	I, AV	3	Fs	0.2	0.06	41.3
Falconiformes							
**Falconidae**							
*Caracara plancus*	Carcará ^i^	I, AV	8	CH, Fs, Pr	0.5	0.25	51.3
*Herpetotheres cachinnans*	Acauã/cauã	I	11	Nc, Sg	0.7	0.31	51
*Falco sparverius*	Quiriquiri	AV	–	–	–	–	–
Psittaciformes							
**Psittacidae**							
Generic term	Araras	I	10	Ca, Nc, CH, C, Se	0.6	0.38	81.3
*Brotogeris chiriri*	Xereré ^i^	I, AV	9	Ca	0.6	0.56	41.3
*Amazona aestiva* ^EN^	Verdadeiro	I, AV	7	Ca, Nc, Fs	0.4	0.44	51.3
*Forpus xanthopterygius*	Periquito	I	6	Ca	0.4	0.38	41.3
*Anodorhynchus hyacinthinus* ^VU^	Arara-azul/preta ^d^	I	4	Ca, Fs, C	0.3	0.25	61.3
*Primolius maracana* ^EN^	Maracanã ^i^	I, AV	16	Nc, Pr	1	0.88	51.3
*Ara ararauna*	Arara-amarela	I	5	Ca, Fs, C, Se, Pr	0.3	0.31	81.3
*Ara chloropterus*	Arara-vermelha	I	3	Ca, Fs, C	0.3	0.25	61.3
Passeriformes							
**Dendrocolaptidae**							
*Lepidocolaptes angustirostris*	Arapaçu-de-cerrado	MN	–	–	–	–	–
**Furnariidae**							
*Furnarius rufus*	João-de-barro	I, AV, MN	6	Nc, Se	0.4	0.38	51.3
*Pseudoseisura cristata*	Carrega-madeira	AV	–	–	–	–	–
**Tityridae**							
*Pachyramphus viridis*	Bico-grosso	MN	–	–	–	–	–
**Rhynchocyclidae**							
*Tolmomyias flaviventris*	Bico-chato-amarelo	MN	–	–	–	–	–
*Hemitriccus margaritaceiventer*	Sebinho-de-olho-de-ouro	MN	–	–	–	–	–
**Tyrannidae**							
*Pitangus sulphuratus*	Bem-te-vi	I, AV	2	Nc	0.1	0.13	41.3
*Knipolegus franciscanus*	Corroxó ^i^	I	4	Nc	0.3	0.13	41.3
*Tyrannus melancholicus*	Suiriri	AV, MN	–	–	–	–	–
**Corvidae**							
*Cyanocorax cyanopogon*	Pêga-branca	I, AV	4	Ca, Sg	0.3	0.25	51.3
**Hirundinidae**							
*Progne tapera*	Andorinha	I, AV	1	Nc	0.1	0.06	41
**Troglodytidae**							
*Troglodytes musculus*	Garrincha	I, AV, MN	2	0	0.1	0.13	41.3
**Turdidae**							
*Turdus rufiventris*	Sabiá-laranja	I, AV	6	Ca	0.4	0.38	41.3
*Turdus leucomelas*	Sabiá-barranco	AV	–	–	–	–	–
**Mimidae**							
*Mimus saturninus*	Papa-cebo ^i^	I, AV	2	Ca	0.1	0.13	41.3
**Passeridae**							
*Passer domesticus*	Pardal ^i^	I, AV	1	Nc, Pr	0.1	0.06	41.3
**Fringillidae**							
*Euphonia chlorotica*	Vim-vim	I	1	Ca	0.1	0.06	41.3
**Passerellidae**							
*Zonotrichia capensis*	Salta-chão	I	2	Ca	0.1	0.13	41.3
**Icteridae**							
*Cacicus solitarius*	Iraúna	I	1	Ca	0.1	0.06	41.3
*Icterus pyrrhopterus*	Pêga-de-banana	I, AV	1	Nc	0.1	0.06	41.3
*Icterus jamacaii*	Sofreu ^i^	I, AV	4	Nc, C	0.3	0.38	51.3
*Molothrus rufoaxillaris*	Currutié ^i^	I, AV	4	Ca, Nc	0.1	0.06	41.3
*Molothrus bonariensis*	Currute ^i^	I, AV, MN	2	Nc	0.1	0.13	41.3
*Gnorimopsar chopi*	Pássaro-preto ^i^	I	6	Ca, CH	0.4	0.38	51.3
*Chrysomus ruficapillus*	Golinha-vermelha	I, AV, MN	1	Nc	0.3	0.25	51.3
**Cardinalidae**							
*Cyanoloxia brissonii*	Azulão	I	2	Ca	0.1	0.13	41.3
*Saltatricula atricollis*	Bico-amarelo	I, AV	2	0	0.1	0	31.3
**Thraupidae**							
*Saltator fuliginosus*	Bico-de-pimenta ^i^	I, AV	1	Ca	0.1	0.06	41.3
*Coryphospingus pileatus*	Tico-tico-rei-cinza	MN	–	–	–	–	–
*Ramphocelus bresilia*	Sangue-de-boi ^i^	I	2	Ca	0.1	0.13	51.3
*Sporophila lineola*	Bigode	I, AV	2	Ca	0.1	0.13	41.3
*Sporophila plumbea*	Patativa	I, MN	2	Ca	0.1	0.13	41.3
*Sporophila nigricollis*	Chupa	I, AV, MN	4	Ca, C	0.3	0.25	51.3
*Sporophila caerulescens*	Coleira	I, AV, MN	6	Ca	0.4	0.06	41.3
*Sporophila angolensis*	Curió ^i^	I	3	Ca, C, Se	0.2	0.19	61.3
*Sicalis flaveola*	Canário-da-terra^d^	I, AV, MN	8	Ca, CH, C	0.5	0.5	61.3
*Sicalis columbiana*	Xuré ^i^	I, AV, MN	22	Ca, CH, C	1.4	1	61.3
*Paroaria dominicana*	Cabeça-vermelha ^d^	I, AV, MN	9	Ca, C	0.6	0.31	51.3
*Thraupis sayaca*	Sanhaço	I, AV, MN	4	Nc	0.3	0.13	41.3
*Thraupis palmarum*	Sanhaço-coqueiro	AV	–	–	–	–	–

Sampling: Interviews (I), Audiovisual (AV), Mist Net (MN). Total Number of Citations for categories (TNC). Use’s Categories of the species according to their utilitarian by the community: Captivity for affection (Ca), Food source (Fs), Non-captive birds (Nc), Signals (Sg), Capture/Hunting (CH), Commercial (C), Benefits (Be), Prejudice (Pr) and, Not assigned (0). Ethnobiological indices: Relative Frequency of Citations (RFC). Use Value (UV), Relative Importance (RI); VulnerableVU [62, 88]; EndangeredEN [63, 89]. Ethnospecies highlighted by participants in that: populations decreased d and increased i. Species not cited by participants, category not assigned, indices not calculated (–)

### Implications for biocultural conservation/ethnoecological management

The interactions between capture, handling, captivity, and localized commercialization observed in Santa Rosa also point to broader biocultural implications that extend beyond conservation status alone. Close and prolonged contact between humans and birds, particularly under informal management conditions, has been recognized as a potential interface for zoonotic exposure in other contexts [83, 84]. Although no health-related assessments were conducted in the present study, the practices documented here highlight pathways through which public health, cultural practices, and wildlife management may intersect. From a precautionary perspective, these interfaces reinforce the importance of integrating health awareness into biocultural conservation strategies, especially in regions where bird captivity remains socially normalized and weakly regulated.

Although no confirmed cases of interspecific hybridization were recorded during fieldwork, participant narratives describing the co-keeping of congeneric taxa under captive conditions indicate a biologically plausible risk. Such situations may increase the likelihood of accidental or intentional cross-breeding, particularly where species share similar reproductive behaviors or housing conditions [85, 86]. In Brazil, interspecific hybridization in captivity is explicitly prohibited under federal regulation (IBAMA, IN nº 10/2011, Art. 37), and the presence of hybrid individuals poses challenges for enforcement, species identification, and genetic integrity [86–88]. Within a biocultural framework, these risks underscore the need to reconcile regulatory norms with culturally embedded practices, favoring dialogue and adaptive management over purely punitive approaches.

### Limitations of the study

We acknowledge several limitations in this study that contextualize our findings. First, the absence of official historical and sociodemographic data for the community constrains our baseline comparisons. Second, regarding sampling effort, although data saturation was reached in the interviews, the small sample size (*n* = 16) presents demographic biases (e.g., predominance of male participants, age, and education imbalances), which may constrain the generalization of our findings to the broader population. Furthermore, logistical field constraints limited our ability to conduct more comprehensive birdlife sampling, particularly concerning seasonal variations and reproductive patterns. The use of mist nets coincided with another ongoing project involving sanitary analyses of birds in federal protected areas, making their deployment particularly opportune for this study. This likely resulted in an underestimation of the total local species richness, as the rarefaction curves for abundance (Chao1) did not reach complete stabilization. This environmental subsampling also hinders the analysis of robust correlations between species use-citations and their actual environmental availability (e.g., testing the Ecological Apparency Hypothesis). Thus, our ecological inferences depended primarily on the qualitative depth of the participants’ narratives.

## Conclusion

The empirical patterns observed in Santa Rosa illustrate how conservation conflicts emerge from social and value-based asymmetries, particularly when anthropocentric or protectionist policies fail to align with cultural logics, daily practices, and livelihood needs [8, 89]. Conservation success therefore depends not only on ecological outcomes but also on strengthening community well-being, purpose, autonomy, identity, and cohesion, which underlie the community’s relationship with the landscape and fauna. Interventions that overlook these biocultural dimensions risk being perceived as unfair, reproducing mistrust and resistance toward conservation authorities [90].

Building on these insights, we propose a pragmatic and community-centered strategy to reconcile biocultural continuity with conservation goals: (1) formally recognize and incorporate TEK into local conservation planning and legal outreach; (2) implement culturally acceptable, non-lethal conflict-mitigation and demand-reduction measures (education, deterrents, defensive agronomic practices, incentives); and (3) develop participatory monitoring and livelihood alternatives, such as regulated birdwatching or community-based ecotourism, that redirect cultural valuation from extractive practices toward stewardship. These recommendations arise directly from the dynamics documented in Santa Rosa and surrounding semiarid regions, indicating that ethno-ornithological knowledge can shift from a risk factor to a conservation asset when governance frameworks embrace equitable and bioculturally grounded approaches. Future research should evaluate the ecological efficacy and social acceptability of locally devised management tools, further advancing integrated conservation in semiarid socioecological systems.

## Data Availability

The datasets generated and/or analyzed under license during the current study are not publicly available to protect and not reveal the privacy of the study participants. However, any type of data that does not violate the anonymity of the respondent can be obtained from the corresponding author upon reasonable request.

## Abbreviations

CAAE: Certificate of Presentation of Ethical Appreciation
CEP: Committee of Ethics in Research
FICT: Free and Informed Consent Term
IBAMA: Brazilian Institute of the Environment and Renewable Natural Resources
IUCN: International Union for Conservation of Nature
RFC: Relative Frequency of Citations
TEK: Traditional Ecological Knowledge
UV: Use Value
